# Case report of ectopic hepatic tissue, a rare finding during a laparoscopic cholecystectomy

**DOI:** 10.1016/j.ijscr.2020.01.014

**Published:** 2020-01-23

**Authors:** Afrim Avdaj, Sadie Namani, Anila Cake, Agron Bytyqi

**Affiliations:** a General Hospital “Prim.Dr.Daut Mustafa”, Sheh Emini Street, Prizren, Kosovo; b Clinic of Infectious Diseases, University Clinical Centre of Kosovo, Mother Theresa Street, Hil-Mosi 3A., Prishtina, Kosovo; c Albania and University “Aleksander Xhuvani” Faculty of Nursing in Elbasan, Albania; d Department of Continuing Professional Education, General Hospital “Prim.Dr.Daut Mustafa”, Prizren, Kosovo

**Keywords:** Ectopic hepatic, Gallbladder, Laparoscopy

## Abstract

**Introduction:**

Ectopic hepatic tissue is due to an uncommon failure of embryological liver development. The incidence of ectopic liver has been reported to be anywhere from 0.24% to 0.47% and a prevalence rate of 0.47% as diagnosed at laparotomy or laparoscopy. We report a case of Ectopic Hepatic tissue attached to the gallbladder wall that was discovered during a laparoscopic cholecystectomy.

**Case report:**

A 47 year-old women presented to the surgery department with abdominal acute pain. The patient was taken for a standard laparoscopic cholecystectomy. The gallbladder was retracted cephalad while Hartmann’s pouch was retracted laterally. It was then noted that an ectopic tissue was present on the gallbladder wall. The patient recovered well after surgery, had no complications and was discharged the day after surgery.

**Discussion:**

Ectopic hepatic tissue is a rare condition. The real incidence of ectopic hepatic tissue attached to the gallbladder wall is difficult to assess but is reportedly 0.24–0.47% of the population. Ectopic hepatic tissue attached to the gallbladder usually remains asymptomatic and is occasionally discovered during laparoscopy, as was the case with the patient in the present report. In this case presented, the histopathological examination of specimen was confirmed to be ectopic liver tissue without hepatocellular carcinoma.

**Conclusion:**

It is important to be vigilant of ectopic hepatic tissue, their complications, and the potential surgical, including increased operative time and the need to follow up on such patients to rule out any possible complications.

## Highlights

• Ectopic hepatic tissue is due to an uncommon failure of embryological liver development.
• Ectopic hepatic tissue attached to the gallbladder usually remains asymptomatic and is occasionally discovered during laparoscopy.
• In this case presented, the histopathological examination of specimen was confirmed to be ectopic liver tissue without hepatocellular carcinoma.
• It is important to be vigilant of ectopic hepatic tissue, because of their possible complication.

## 1 Introduction

Ectopic hepatic tissue is due to an uncommon failure of embryological liver development [1]. It is a rare entity that involves the presence of hepatic tissue in a number of sites outside of the native liver such as the gallbladder, hepatic ligaments, omentum, retroperitoneum, and thorax [2,3]. The incidence of ectopic liver has been reported to be anywhere from 0.24% to 0.47% and a prevalence rate of 0.47% as diagnosed at laparotomy or laparoscopy [2,4]. The possibility of malignant transformation into hepatocellular carcinoma and the possible differential diagnoses of gallbladder wall masses make ectopic hepatic tissue challenging for surgeons [5]. We report a case of Ectopic Hepatic tissue attached to the gallbladder wall that was discovered during a laparoscopic cholecystectomy for gallstones. The work has been reported in line with the SCARE criteria [6].

## 2 Case report

**Fig. 1:**
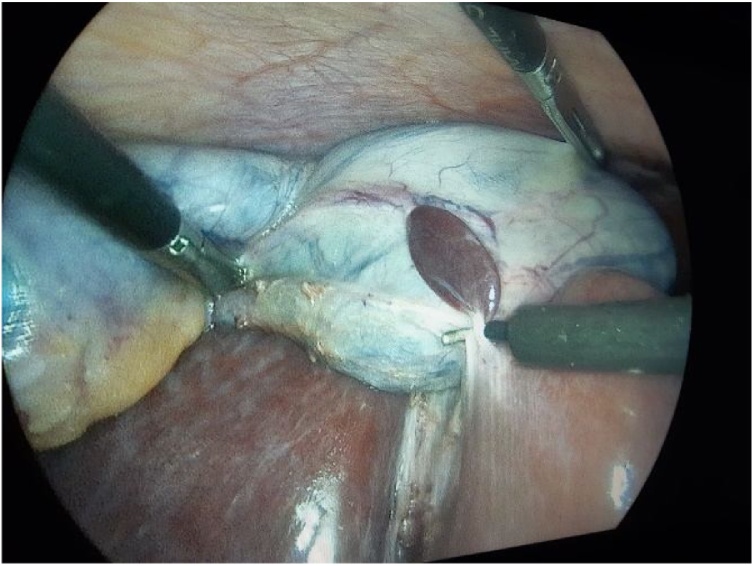
Laparoscopic view of ectopic liver tissue attached to the gallbladder wall.

**Fig. 2:**
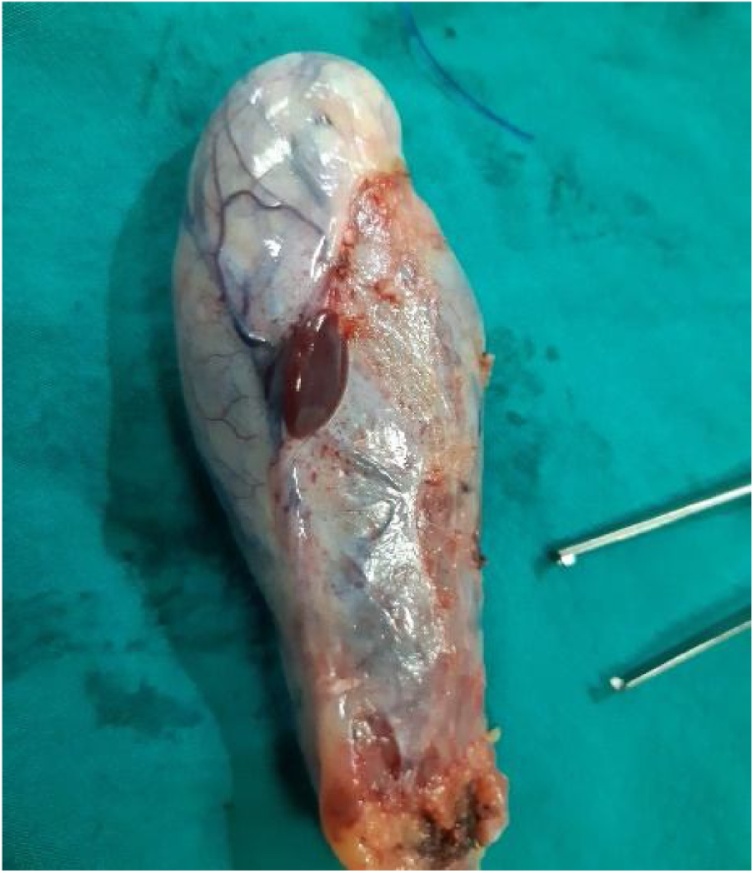
Gross image of resected specimen with ectopic liver tissue attached on the gallbladder.

**Fig. 3:**
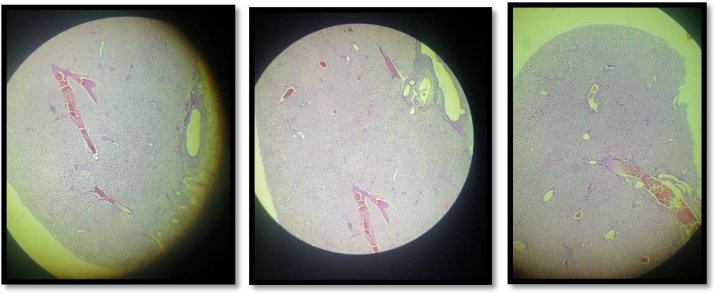
Histopathological microscopy showing the presence of ectopic hepatic tissue without pathological features.

A 47 year-old women presented to the surgery department with abdominal acute pain. During anamnesis, the patient indicates that the pain began nine days ago, with an increased intensity of pain. Occasional complaints have had the last two years with periods of pain relief. When the pain was reported, she was notified by her doctor, treated symptomatically and referred to the surgeon. The next day was reported in the emergency of Prizren General Hospital with acute pain. In an emergency they did the necessary examinations where she was diagnosed with chronic calculous cholecystitis. She was treated with intravenous therapy and was then admitted to the surgery ward at Prizren General Hospital under the care of surgeon. The patient had no significant past medical history and previous surgical intervention. Vital signs were in normal range, TA 145/90 mmHg, T° 37.4 °C. The laboratory evaluation of patient were also in normal range. Abdominal ultrasound scan showed multiples gallstones without evidence of acute cholecystitis and a 3 cm localized thickening of the anterior portion of the gallbladder wall. The patient was taken for a standard laparoscopic cholecystectomy. The abdominal cavity was entered using Hasson’s technique, CO_2_ insufflated to 17 mmHg. Four additional ports were created: one port 10 mm to the right of falciform ligament, one 10 mm port in the subxiphoid region, and two 5 mm ports in the right upper quadrant and ﬂank, respectively. The gallbladder was retracted cephalad while Hartmann’s pouch was retracted laterally. It was then noted that an ectopic tissue was present on the gallbladder wall (Fig. 1, Fig. 2). First the gallbladder was mobilized using the standard procedure. Calot’s triangle was dissected, the cystic artery was clipped and incised. Electrocoagulation was then used to remove the gallbladder from the liver. Careful dissection was done to prevent damage to the ectopic tissue with the electrocautery. The specimen was removed via endo pouch and sent for histopathological examination. The tissue was confirmed to be ectopic liver (Fig. 2, Fig. 3). The patient recovered well after surgery, had no complications and was discharged the day after surgery.

## 3 Discussion

Ectopic hepatic tissue is a rare condition [7]. The real incidence of ectopic hepatic tissue attached to the gallbladder wall is difficult to assess but is reportedly 0.24–0.47% of the population though as with other sites, most of the cases of ectopic hepatic tissue attached to the gallbladder are diagnosed at laparotomy, laparoscopy or during an autopsy. Even though the gallbladder is the main site for the development of ectopic hepatic tissue, this finding is still exceptional [2,4].

Ectopic hepatic tissue is divided into four distinct categories: (a) Ectopic hepatic tissue that is not connected to the main liver and is usually attached to the gallbladder or intra-abdominal ligaments; (b) microscopic ectopic liver found occasionally in the gallbladder wall (as it was in the case presented here); (c) a large accessory liver attached to the “mother” liver by a stalk; and (d) a small accessory liver lobe attached to the main liver [5].

Ectopic hepatic tissue is sometimes associated with other congenital anomalies, such as biliary atresia, agenesis of the caudate lobe, omphalocele, bile duct cysts or cardiac anomalies; however, these abnormalities are not present when the heterotopic tissue is attached in the surface of gallbladder wall [8]. Ectopic hepatic tissue attached to the gallbladder usually remains asymptomatic and is occasionally discovered during laparoscopy, as was the case with the patient in the present report. When symptomatic, the principal complaint is usually upper abdominal pain due to complications such as torsion, hemorrhagic necrosis, rupture or some form of compression by the mass due to malignant transformation to hepatocellular carcinoma. The differential diagnosis of lesions attached to the gallbladder includes other diseases that lead to a mass effect, such as carcinoma of the gallbladder, polyps, accessory liver, adenomyomatosis, hyperplastic lymph nodes and metastatic disease [2,7,[9], [10], [11]].

Detection of ectopic hepatic tissue before surgical intervention or autopsies by means of imaging studies is rare [2,12]. This may be due to the small size of most ectopic hepatic tissue, the lack of awareness of this unusual condition among radiologists, difficulty interpreting the imaging and the frequent lack of symptoms [12]. The diagnosis of ectopic hepatic tissue should be considered when radiologists identify a soft tissue mass on the gallbladder wall on imaging (whether ultrasound, CT scan, or MRI), or as an incidental finding during laparoscopy. Percutaneous biopsies should be avoided because of the risk of bleeding and the possibility of malignant degeneration to hepatocellular carcinoma. In the patient described in the present report, the abdominal ultrasonographic examination before surgery showed multiples gallstones without evidence of acute cholecystitis and a slight thickening of the anterior wall of the gallbladder, insufficient to suggest ectopic hepatic tissue.

The complications of an ectopic liver include torsion, peritoneal bleeding, fatty change with evolution to cirrhosis, and malignant degeneration to hepatocellular carcinoma [1]. There has been evidence to suggest that ectopic liver is at increased risk of developing hepatocellular carcinoma [8,13]. Arakawa et al. [14] found 21 cases in the literature, mostly from Japan, of hepatocellular carcinoma arising extrahepatically. These authors suggest that ectopic tissue is more susceptible to the development of malignancy because it does not have a complete vasculature or ductal system like a normal liver, and is perhaps functionally impaired. This altered hepatic function may lead to chronic inflammation or cirrhosis, which increases the possibility of developing hepatocellular carcinoma. Yamashita et al. [15] reviewed 70 cases of ectopic liver reported in the literature before 1986, including nine cases of hepatocellular carcinoma originating in ectopic hepatic tissue. Of 48 cases (excluding those localized to the gallbladder), 22 developed hepatocellular carcinoma [14], contrasting with only one of 42 cases of ectopic hepatic tissue attached to the gallbladder. A possible explanation for this difference is that ectopic hepatic tissue attached to the gallbladder is an anomaly occurring later during the development of the biliary bud and is therefore well differentiated [8]. Despite the presence of steatosis and areas of hemosiderosis, we could not find any evidence of malignant degeneration in the patient in this report. However, due to the perceived risk of malignant degeneration in ectopic hepatic tissue generally, resection of the ectopic hepatic tissue en bloc with the gallbladder is strongly recommended [12]. With the increase in laparoscopic cholecystectomy, more cases of ectopic hepatic tissue attached to the gallbladder have been identified in recent years and are easily removed using this approach, preferably inside a bag and then carefully examined by a pathologist. If the histopathological examination confirms the presence of an invasive hepatocellular carcinoma, a further surgical intervention should be considered, to extend the hepatic margins of resection and perform a regional lymphadenectomy [1]. In this case presented, the histopathological examination of specimen was confirmed to be ectopic liver tissue without hepatocellular carcinoma.

As conclusion we have to emphasize the importance of being vigilant of ectopic hepatic tissue, their complications, and the potential surgical risks as described above, including increased operative time and the need to follow up on such patients to rule out any possible complications. With the limited literature available ectopic hepatic tissue can be missed on radiological exam, as in our case. Since cholecystectomy is a routinely performed surgery, surgeon awareness will help combat any potential intraoperative complications.

## Funding

None.

## Ethical approval

The article was approved by Ethical Committee of General Hospital of Prizren (process No. 7/242).

## Consent

Written informed consent was obtained from the patient for publication of this case report and accompanying images.

## Author contribution

Afrim Avdaj contributed toward surgical team, writing and final revision.

Sadije Namani contributed toward data analysis and writing.

Anila Cake contributed toward review of the literature.

Agron Bytyqi contributed toward study design, data collections, writing and data analysis.

## Registration of research studies

Not applicable.

## Guarantor

Afrim Avdaj.

## Provenance and peer review

Not commissioned, externally peer-reviewed.

## Declaration of Competing Interest

There is no conflict of interests between the authors regarding the publication of this article.
